# Subjective quality of life in war-affected populations

**DOI:** 10.1186/1471-2458-13-624

**Published:** 2013-07-02

**Authors:** Aleksandra Matanov, Domenico Giacco, Marija Bogic, Dean Ajdukovic, Tanja Franciskovic, Gian Maria Galeazzi, Abdulah Kucukalic, Dusica Lecic-Tosevski, Nexhmedin Morina, Mihajlo Popovski, Matthias Schützwohl, Stefan Priebe

**Affiliations:** 1 Unit for Social and Community Psychiatry, Barts and The London School of Medicine and Dentistry, Queen Mary University of London, Cherry Tree Way, London E13 8SP, United Kingdom; 2 Unit for Social and Community Psychiatry, Barts and The London School of Medicine and Dentistry, Queen Mary University of London, London, United Kingdom; 3 Unit for Social and Community Psychiatry, Barts and The London School of Medicine and Dentistry, Queen Mary University of London, London, United Kingdom; 4 Department of Psychology, Faculty of Humanities and Social Sciences, University of Zagreb, Zagreb, Croatia; 5 School of Medicine, University of Rijeka, Rijeka, Croatia; 6 Department of Psychiatry, University of Modena and Reggio Emilia, Modena, Italy; 7 School of Medicine, University of Sarajevo, Sarajevo, Bosnia and Herzegovina; 8 Belgrade University School of Medicine, Belgrade, Serbia; 9 Department of Clinical Psychology, University of Amsterdam, Amsterdam, Netherlands; 10 Faculty of Philosophy, University of Skopje, Skopje, FYR Macedonia; 11 Department of Psychiatry and Psychotherapy, University Hospital Carl Gustav Carus, Technische Universität Dresden, Dresden, Germany; 12 Unit for Social and Community Psychiatry, Barts and The London School of Medicine and Dentistry, Queen Mary University of London, London, United Kingdom

**Keywords:** Subjective Quality of Life, War Trauma, Post-conflict Settings, Refugees

## Abstract

**Background:**

Exposure to traumatic war events may lead to a reduction in quality of life for many years. Research suggests that these impairments may be associated with posttraumatic stress symptoms; however, wars also have a profound impact on social conditions. Systematic studies utilising subjective quality of life (SQOL) measures are particularly rare and research in post-conflict settings is scarce. Whether social factors independently affect SQOL after war in addition to symptoms has not been explored in large scale studies.

**Method:**

War-affected community samples were recruited through a random-walk technique in five Balkan countries and through registers and networking in three Western European countries. The interviews were carried out on average 8 years after the war in the Balkans. SQOL was assessed on Manchester Short Assessment of Quality of Life - MANSA. We explored the impact of war events, posttraumatic stress symptoms and post-war environment on SQOL.

**Results:**

We interviewed 3313 Balkan residents and 854 refugees in Western Europe. The MANSA mean score was 4.8 (SD = 0.9) for the Balkan sample and 4.7 (SD = 0.9) for refugees. In both samples participants were explicitly dissatisfied with their employment and financial situation. Posttraumatic stress symptoms had a strong negative impact on SQOL. Traumatic war events were directly linked with lower SQOL in Balkan residents. The post-war environment influenced SQOL in both groups: unemployment was associated with lower SQOL and recent contacts with friends with higher SQOL. Experiencing more migration-related stressors was linked to poorer SQOL in refugees.

**Conclusion:**

Both posttraumatic stress symptoms and aspects of the post-war environment independently influence SQOL in war-affected populations. Aid programmes to improve wellbeing following the traumatic war events should include both treatment of posttraumatic symptoms and social interventions.

## Background

Exposure to war has been associated with lower quality of life (QOL) even after the end of the actual hostilities [1-3]. The effects of war-related events may persist for many years [4,5]. Research has established a high prevalence of mental disorders in war-affected populations, in particular posttraumatic stress disorder (PTSD) and depression [6,7]. QOL deficits in both veterans and refugees have been consistently linked with war-related posttraumatic stress symptoms [8-10]. However, wars and armed conflicts also cause lasting changes in social conditions through increased poverty, lack of employment, community violence, inadequate living circumstances and changed social networks [11,12]. The associations between post-war mental distress, social environment and QOL have not been systematically explored in large scale studies. Existing studies were mostly carried out on samples of war veterans and refugees in the West, and it is not clear whether findings can be applied to post-conflict settings. Systematic research utilising subjective quality of life measures in war-affected populations is particularly rare.

The concept of QOL has been introduced into mental health research in order to account for the effects of both mental illness and relevant interventions on the broader context of patients’ lives. It is linked to the WHO definition of health in general as a state of complete physical, mental and social-wellbeing, and not merely absence of disease and infirmity [13]. Different theoretical frameworks have been used to define the concept; however, there has been a growing trend towards capturing individuals’ own understandings [9]. Subjective quality of life (SQOL) has become an established patient -reported outcome in recent years [14]. The concept reflects subjective appraisal of life and life conditions and uses mainly satisfaction constructs [15].

The collapse of Yugoslavia in the early 1990s precipitated the worst armed conflict in Europe since WWII, with war activities spanning between 1991 and 2001. The CONNECT study assessed mental health consequences of the war in both people who reside in the countries of the region and those who were resettled in Western Europe [16,17]. The participants experienced at least one war-related potentially traumatic event and lived in areas that had been directly exposed to war activities. On average 8.6 years had elapsed since the occurrence of the most distressing war events. Mental disorders were found to be highly prevalent, with 33.5% and 28.3% of respondents reporting anxiety (including PTSD) and mood disorders in the Balkan sample, while these figures were 43.7% and 43.4% respectively amongst the refugees. The aim of the present analysis was to assess overall SQOL as well as the satisfaction with individual life domains. The associations with exposure to traumatic war events, posttraumatic stress symptoms and post-war environment were explored. To our knowledge this is the largest study to date assessing SQOL and associated factors in both residents of post-conflict regions and in compatriot refugees in the West.

## Methods

The data was collected within the CONNECT project, a multi-centre epidemiological study, the aim of which was to assess mental health consequences of war and migration, and associated use of health and social care services [18]. The assessments were carried out between January 2005 and November 2006 across five Balkan countries: Croatia, Serbia, Bosnia and Herzegovina, FYR Macedonia and Kosovo; and amongst refugees in three Western European countries: Germany, Italy and the United Kingdom.

### Sampling techniques and participants

Participants in Balkan countries were recruited using a multi-stage probabilistic sampling frame and random-walk approach in administrative regions that had been directly exposed to war activities. Random-walk is door-to-door survey technique for systematically gathering a random sample of households after starting at a particular point. The technique is often used in post-emergency settings when complete data on the affected population is still not available [19,20]. Health surveys combining a cluster sampling design and random-walk have been widely used across developing countries [21] and evaluated in different contexts [22,23]. In this particular study we randomly selected a total of 15 war-affected regions across five countries, and, within them, a further 49 localities. Within each locality streets were randomly identified and every fourth household approached for participation in the study. We assessed an adult member of each chosen household whose birthday was closest to the date of interviewing.

Refugees from the Balkans who reside in Western Europe were assessed in Germany, Italy and the UK. These countries were chosen due to the high numbers of immigrants they received from former Yugoslavia in the 1990s. Since the application of sampling techniques used in the Balkans was not feasible in the West, refugees were recruited through a combination of random and non-random sampling approaches. In Germany and Italy, potential interviewees were identified through resident registers and snowball sampling. Potential participants in the UK were contacted through community organisations and snowball sampling, as the relevant registers were not available.

Participants were included if they had been born within the territory of former Yugoslavia; were between 18 and 65 years old; had experienced at least one war-related potentially traumatic event (to ensure that all participants had been-affected by war); had experienced the most recent war-related event at 16 years of age or older; had no severe learning difficulty and no mental impairment due to brain injury or other organic causes. The details of sampling techniques and characteristics of the sample have been described in previous publications [16,17].

### Procedures and measures

Participants’ socio-demographic characteristics including age, sex, marital status, educational level, current employment status and accommodation were obtained using a brief structured questionnaire.

Potentially traumatic experiences before, during and after the war were assessed on an adapted 24 item version of the Life Stressor Checklist-Revised [10,24]. Cumulative scores for pre-war, war, and post-war experiences were calculated.

The Manchester Short Assessment of Quality of Life - MANSA was used to assess SQOL [25]. The instrument contains twelve items which assess satisfaction with life as a whole and across different life domains (life in general, employment, financial situation, friendships, leisure activities, accommodation, personal safety, people living in household/living alone, sex life, relationship with family, physical and mental health). The items are assessed on a 7 point Likert scale (1–7). The underlying concept of quality of life is generic: all questions allow comparison with the general population and are not specifically illness- or symptom-related. The instrument was used previously on war-affected population in the Balkans and refugees from the region resettled in the West [10].

Levels of posttraumatic stress symptoms were measured on the Impact of Events Scale-Revised (IES-R) [26]. This self-report instrument assesses 22 intrusion, avoidance and hyperarousal symptoms within the last 7 days in relation to a specific traumatic event. Each IES-R item is rated on a five-point scale of distress (0–4).

Depressive symptoms were assessed on the Brief Symptom Inventory (BSI) [27]. This 53-item self-report instrument is designed to measure nine primary psychological symptom dimensions (somatisation, obsessive-compulsive, interpersonal sensitivity, depression, anxiety, hostility, phobic anxiety, paranoid ideation and psychoticism). Each BSI item is rated on a five-point scale of distress (0–4), ranging from "not at all" (0) to "extremely" (4), in relation to the past seven days. The score is calculated as a mean value of endorsed items, with the range between 0 and 4.

Social contacts were assessed on a single item of the MANSA questionnaire. Participants’ responses to the MANSA item 5 (whether they had met a friend in the previous week) were used as an indicator of having social contacts.

Experiences of displacement during the war were assessed in the Balkan sample (refugees or internally displaced vs. those who remained at home). Participants in the Western European countries were additionally asked about possible post-migration stressors (separation from family, difficulties in obtaining a work permit or work in own profession, financial difficulties, inadequate accommodation, difficulties in accessing medical care) they may have experienced in the host country resulting in a cumulative score of stressors ranging from zero to six.

All assessments were carried out face-to-face by trained interviewers who were native speakers of the languages used in the study (Serbian, Croatian, Bosnian, Macedonian and Albanian), apart from one interviewer in Germany who was bilingual. A detailed description of the study was provided to all potential participants and written informed consent was obtained prior to the interview. All those instruments for which there had been no validated translations in the relevant languages were translated and back-translated into English. The study was approved by the relevant national ethics committees: Ethic Committee for Research with Human Subjects, Faculty of Humanities and Social Science, University of Zagreb; Ethics Committee, School of Medicine, University of Belgrade; Ethics Committee, University of Sarajevo Clinical Center; Ethics Committee, School of Medicine, University of Rijeka; Ethics Committee, Faculty of Philosophy, University of Skopje; Ethics Committee, Technische Universität Dresden; Ethics Committee of Modena Municipality and Royal Free Hospital & Medical School Local Research Ethics Committee.

### Statistical analysis

Descriptive statistics were used to summarise data as counts and percentages, or means and standard deviations as appropriate. Independent sample *t*-test was conducted to compare the scores of individual MANSA items for Balkan and Western European samples.

Linear regression was used to assess the relationship between the explanatory variables and outcome, which was the mean score of SQOL on MANSA. The results are presented as regression coefficients (B) with 95% confidence intervals (CI) and *p*-values. Firstly, we conducted univariable analyses to identify factors significantly associated with the level of SQOL on MANSA. The following candidate variables were included: age; gender (female vs. male); education level (secondary or higher vs. primary or none); marital status (married or cohabiting vs. single, divorced or widowed); count of traumatic events experienced (number of traumatic events in pre-war, war and post-war period), posttraumatic stress symptoms (IES-r); employment (being unemployed vs. employed, retired or in education); accommodation (living in collective accommodation vs. home ownership, renting or living with family and friends); and social contacts (having met a friend in the previous week or not). Additionally, experience of displacement during the war (refugees or internally displaced vs. those who remained at home) was included as a candidate variable for the Balkan sample, and the count of post-migration stressors for the refugee sample. In the next step, those variables that were significant at the 5% level were entered into a multivariable model with the same outcome variable. At this point the analysis was adjusted for the country of residence. We intended to adjust the analysis for the level of depressive symptoms on BSI; however, depressive symptoms showed high multi-collinearity with posttraumatic stress symptoms in both samples (Balkan: r = .597, p < 0.001; refugees r = .649, p < 0.001). We therefore could not consider post-traumatic stress symptoms and depressive symptoms in the same analysis and decided to focus on post-traumatic stress symptoms because of their specific importance for war-related psychological distress.

To address any potential issues arising from missing MANSA values a multiple imputation was carried out and analysis repeated on the pooled data. All variables included in the analysis were entered both as predictors and for imputing. Twenty-five imputations were conducted in both datasets. The results were comparable to those obtained in the original analysis.

The analyses were carried out separately on the samples from the Balkans and the West in order to avoid biases in the interpretation of results. All analyses were performed using SPSS version 18 (SPSS Inc., Chicago, IL, USA).

## Results

In the Balkans we assessed 3313 (70.1%) out of 5330 potential participants, with more than 600 interviewed in each participating country (Croatia: 727; BiH: 640, Kosovo: 648; FYR Macedonia: 661; Serbia: 637). 1414 (26.5%) of participants declined to participate and 603 (11.3%) did not satisfy the inclusion criteria. In Western Europe we interviewed 627 (52.9%) out of 2263 potential participants who were selected through resident registers or community organisations and who responded to the invitation letter. 558 (24.7%) refused to participate and 1078 (47.6%) did not meet inclusion criteria. A further 227 participants were recruited through snowballing, resulting in a total sample of 854 (Germany: 255; Italy: 297; UK: 302).

### The characteristics of participants

The mean age of participants in the Balkan sample was 42.5 (SD = 12) and 53.8% were female. They experienced on average 4.2 (SD = 2.8) war-related traumatic events. The mean age of refugees in the West was 41.6 (SD = 10.8) and 51.3% were women. The average number of war-related traumatic events in this sample was 6.8 (SD = 3.6). Participants in the Balkans sample experienced their most traumatic war event on average 8.1 (SD = 3.3) years prior to interview, while the length of this period for refugees was 10.5 (SD = 3.1) years. Socio-demographic and war-related characteristics of participants from both samples are presented in Table 1.

**Table 1:** Characteristics of participants

**Characteristics**	**Total**	**Balkans**	**Refugees in the West**
	**N = 4167**	**N = 3313**	**N = 854**
Female gender^1^ (N, %)	2221 (53.3)	1783 (53.8)	438 (51.3)
Age (mean years, SD)	42.3 (11.8)	42.5 (12.0)	41.6 (10.8)
Education level (N, %)			
None or primary	1195 (28.7)	1007 (30.4)	188 (22.0)
Secondary	1972 (47.3)	1618 (48.8)	354 (41.5)
Vocational/higher	1000 (24.0)	688 (20.8)	312 (36.5)
Marital status^2^ (N, %)			
Married/cohabiting	2980 (71.5)	2328 (70.3)	652 (76.3)
Single	695 (16.7)	606 (18.3)	89 (10.4)
Divorced/separated/widowed	491 (11.8)	378 (11.4)	113 (13.2)
Employment status (N, %)			
Employed/In training	1714 (41.1)	1329 (40.1)	385 (45.1)
Unemployed	1983 (47.6)	1545 (46.6)	438 (51.3)
Retired	470 (11.3)	439 (13.3)	31 (3.6)
Number of traumatic events (Mean, SD)			
Pre-war traumatic events	0.8 (1.1)	0.7 (1.1)	1.1 (1.3)
War traumatic events	4.7 (3.2)	4.2 (2.8)	6.8 (3.6)
Post-war traumatic events	0.7 (0.9)	0.6 (0.8)	1.1 (1.3)
Living in collective accommodation^3^	322 (7.7)	282 (8.5)	40 (4.7)
Had met a friend in a week before interview^4^	3378 (81.1)	2676 (80.8)	702 (82.2)
Experienced war-related displacement^5^	-	1486 (44.9)	854 (100)
Number of migration stressors (Mean, SD)	-	-	2.6 (1.6)
**Posttraumatic stress (Mean, SD)**	1.2 (1.1)	1.1 (1.1)	1.5 (1.2)

^1-5^Missing values: 1:1; 2:1; 3:1; 4:17; 5:7.

### Subjective quality of life

The MANSA mean score was 4.8 (SD = 0.9) for the Balkan sample, and 4.7 (SD = 0.9) for refugees in Western Europe, indicating similar values above the middle point of 4.0 on the rating scales. The values of the MANSA mean score as well as the scores for individual life domains are presented in Table 2.

**Table 2:** Manchester Short Assessment Of Quality of Life (MANSA): total mean score and individual life domain scores

**MANSA**	**Total sample**	**Balkans**	**Refugees in the West**	***t*-test**
	**N = 4158**	**N = 3313**	**N = 845**	***p***
Total score, mean (SD)	4.78 (0.9)	4.80 (0.9)	4.73 (0.9)	0.67
**Individual items, mean (SD)**				
Life in general^1^	4.4 (1.4)	4.4 (1.4)	4.5 (1.3)	.181
Employment/retirement^2^	3.7 (1.7)	3.6 (1.8)	3.8 (1.7)	.006
Financial situation^3^	3.4 (1.6)	3.3 (1.6)	3.9 (1.5)	< 0.001
Friendships^4^	5.0 (1.3)	5.1 (1.3)	4.8 (1.4)	< 0.001
Leisure activities^5^	4.4 (1.5)	4.4 (1.5)	4.1 (1.5)	< 0.001
Accommodation^6^	4.9 (1.5)	4.9 (1.5)	5.0 (1.5)	.134
Personal safety^7^	5.1 (1.3)	5.1 (1.3)	5.1 (1.4)	.995
People living with/living alone^8^	5.7 (1.2)	5.7 (1.2)	5.8 (1.4)	.256
Sex life^9^	5.0 (1.6)	5.1 (1.6)	4.9 (1.7)	.037
Relationship with family^10^	5.8 (1.1)	5.8 (1.1)	5.9 (1.2)	.097
Physical health^11^	4.9 (1.5)	4.9 (1.5)	4.6 (1.6)	< 0.001
**Mental Health**^**12**^	5.1 (1.6)	5.3 (1.5)	4.6 (1.8)	< 0.001

^1-12^Missing values for individual MANSA items: 1:21; 2:19; 3:16; 4:14; 5:16; 6:15; 7:16; 8:12; 9:382; 10:13; 11:12; 12:10.

There were clear differences in satisfaction ratings across different life domains within each group. In both samples participants were relatively satisfied with their family, household members, personal safety, friendships, sex life, accommodation, and their mental and physical health. However, they were explicitly dissatisfied with their financial and employment situation.

Independent sample t-tests comparing the scores of single MANSA items between two samples revealed some significant differences. Participants in the Balkans were more satisfied with their friendships, leisure activities, sex life, and their physical and mental health. However, they were less satisfied with their employment status and financial situation than refugees in Western Europe. Both groups reported a similar level of satisfaction with life in general, family, household members living alone, accommodation and personal safety.

### Factors associated with subjective quality of life

#### Balkan sample

The results of the univariable and multivariable regression analyses for the Balkan sample are presented in Table 3. Checks for multi-collinearity of predictor variables were satisfactory, with all tolerance values above 0.5 and VIF values below 2.

**Table 3:** Univariable and multivariable linear regression analyses with MANSA life satisfaction score as a dependent variable*

	**BALKAN SAMPLE**					
**N = 3313**	**Univariable regression**			**Multivariable regression**		
	**B**	**95% (CI)**	***p-value***	**B**	**95% (CI)**	***p-value***
SOCIO-DEMOGRAPHICS						
Gender						
Male	.009	-.053 to .072	.769			
Female	0					
Age	-.017	-.019 to -.014	< 0.001	-.012	-.014 to -.009	< 0.001
Education						
Primary or none	0			0		
Secondary	.154	.083 to .225	< 0.001	-.026	-.090 to .038	.431
Higher	.443	.355 to .530	< 0.001	.131	.050 to .213	.002
Being married						
Yes	.142	.074 to .210	< 0.001	.222	.165 to .280	< 0.001
No	0			0		
TRAUMATIC EVENTS						
Pre-war traumatic events	-.112	-.141 to -.083	< 0.001	-.034	-.060 to -.009	.009
War-related traumatic events	-.056	-.067 to -.045	< 0.001	-.026	-.038 to -.013	< 0.001
Post-war events traumatic events	-.142	-.181 to -.103	< 0.001	-.051	-.084 to -.018	.002
Displacement	0.46	-.016 to .109	.148			
PSYCHIATRIC SYMPTOMS						
Posttraumatic stress symptoms	-.377	-.404 to -.351	< 0.001	-.315	-.341 to -.288	< 0.001
POST-WAR ENVIRONMENT						
Being unemployed						
Yes	-.492	-.554 to -.431	< 0.001	-.294	-.350 to -.238	< 0.001
No	0			0		
Living in collective accommodation						
Yes	-.595	-.705 to -.486	< 0.001	-.188	-.282 to -.093	< 0.001
No	0			0		
Having met a friend in the previous week						
Yes	.600	.523 to .676	< 0.001	.493	.427 to .559	< 0.001
**No**	0			0		

*Model adjusted for the country of residence.

In univariable analyses having secondary or higher education, being married and meeting a friend in the previous week were associated with higher levels of SQOL on MANSA. Older age, experiencing more pre-war, war and post-war traumatic events, higher level of posttraumatic stress, being unemployed, and living in collective accommodation were all associated with lower MANSA scores.

In the multivariable model, after adjusting for country of residence, higher education, being married and meeting a friend in the previous week remained associated with higher MANSA scores. Older age, greater number of pre-war, war and post-war traumatic events, higher posttraumatic stress, being unemployed and living in collective accommodation also retained their significant association with lower SQOL.

#### Refugee sample

The results of the univariable and multivariable regression analyses for the sample of refugees resettled in the West are presented in Table 4. Checks for multi-collinearity of predictor variables were satisfactory, with all tolerance values above 0.5 and VIF values below 2.

**Table 4:** Univariable and multivariable linear regression analyses with MANSA life satisfaction score as a dependent variable*

	**REFUGEES IN WESTERN EUROPE**					
**N = 854**	**Univariable regression**			**Multivariable regression**		
	**B**	**95% (CI)**	***p-value***	**B**	**95% (CI)**	***p-value***
SOCIO-DEMOGRAPHICS						
Gender						
Male	.082	-.042 to .206	.194			
Female	0					
Age	-.010	-.016 to -.005	< 0.001	-.002	-.007 to .004	.573
Education						
Primary or none						
Secondary	.112	-.051 to .275	.179			
Higher	.109	-.058 to .276	.199			
Being Married						
Yes	.266	.122 to .411	< 0.001	.313	.188 to .439	< 0.001
No	0			0		
TRAUMATIC EVENTS						
Pre-war traumatic events	-.102	-.148 to -.056	< 0.001	-.080	-.120 to -.039	< 0.001
War traumatic events	-.034	-.051 to -.017	< 0.001	.013	-.003 to .030	.112
Post-war events traumatic events	-.141	-.190 to -.093	< 0.001	-.050	-.094 to -.007	.023
PSYCHIATRIC SYMPTOMS						
Posttraumatic stress symptoms	-.310	-.357 to -.263	< 0.001	-.252	-.304 to -.200	< 0.001
POST-WAR ENVIRONMENT						
Migration stressors	-204	-.241 to -.166	< 0.001	-.127	-.165 to -.089	< 0.001
Unemployed						
Yes	-.381	-.503 to -.259	< 0.001	-.258	-.379 to -.138	< 0.001
No	0			0		
Collective accommodation						
Yes	-.399	-.691 to -.108	.007	-.063	-.321 to .196	.635
No	0			0		
Having met a friend in the previous week						
Yes	.368	.202 to .534	< 0.001	.253	.109 to .396	.001
**No**	0			0		

*Model adjusted for the country of residence.

In univariable analyses being married and meeting a friend in the previous week were the only variables associated with higher SQOL. Older age, more pre-war, war and post-war traumatic events; higher levels of posttraumatic stress, being unemployed and living in collective accommodation were all associated with a lower MANSA score. Reporting more migration-related stressors was also associated with lower SQOL.

In the multivariable model, after adjusting for country of residence, being married and having had a meeting with a friend in the previous week remained positively associated with MANSA scores. Negative associations between, posttraumatic stress, being unemployed, migration stressors and SQOL also retained their significance. The association between war-related traumatic events and MANSA score was not confirmed in the multivariable model, however the significance of pre-war and post-war events was preserved.

## Discussion

### Main findings

This study assessed the level of SQOL in war-affected communities in the Balkans and amongst compatriot refugees in the Western Europe on average eight years after the war. Participants in both groups were explicitly dissatisfied with their employment and financial situation. In contrast, they were more satisfied with their mental and physical health. Satisfaction with family relationships, household members, and personal safety was particularly high. Both war-related posttraumatic stress and socio-economic conditions of the post-war environment were independently associated with levels of SQOL. Almost a decade after the war, posttraumatic stress symptoms still exerted a substantial negative impact on SQOL in both samples. Greater exposure to war-related traumatic events was associated with reduced SQOL in Balkan residents even after controlling for related symptoms. The influence of the post-war environment was important in both groups: recent meetings with friends were associated with higher, and being unemployed with lower MANSA scores. Poorer SQOL was also linked to living in collective accommodation in the Balkans and experiencing more migration-related stressors in the West.

### Strengths and weaknesses

The study used consistent assessment methods across eight countries, and included refugees, civilians and people with combat experience. Standardized instruments were used to measure SQOL and exposure to traumatic events. They were administered face-to-face to participants by trained researchers who were either native speakers of regional languages or bilingual. A multi-stage probabilistic sampling method ensures that the findings are representative for large populations in the war-affected areas.

However, there are also certain limitations to the study. Recruitment in the Western European countries was partly carried out using snowball sampling which may have led to non-representative samples. The representative sample from Balkans and the sample from the West were therefore analysed separately. Finally, the impact of depressive disorders on quality of life was not controlled for due to the high levels of collinearity with posttraumatic symptom scores.

### Comparison with the literature

The MANSA mean score of 4.8 (SD = 0.9) in the representative Balkan sample was higher than the scores obtained previously for war-exposed people from the same region. A study carried out on a community sample of people who suffer from posttraumatic stress reported an average MANSA score of 4.1 (SD 0.9) in Croatia, and 3.9 (SD 0.7) in Serbia [10]. The mean MANSA score of 4.1 (SD 1.1) was found in the sample of Serbian students exposed to the NATO bombing campaign [28]. Similarly, the score of 4.7 (SD = 0.9) reported for the refugees in the West was higher than the MANSA scores indicated by previous findings on refugees resettled in Germany (3.6; SD 1.1) and the UK (4.1; 1.0) [10]. Some of these differences might be explained by the fact that previous research was carried out on selective samples rather than representative ones as in our study.

Participants in both samples, and particularly in the Balkans, were dissatisfied with their employment and financial situation. This finding is unsurprising given the scale of socio-economic instability in post-conflict regions, but also the uncertainties that refugees face in host countries. In contrast, levels of satisfaction with family relationships, household members and personal safety were high, with similar scores obtained in both groups. Overall, participants were more satisfied with their mental and physical health than with their employment and finances. A similar pattern of satisfaction with individual life domains on MANSA was reported by users of community mental health centres in the Balkan countries [29].

With regard to socio-demographic factors influencing SQOL, more education was associated with higher and older age with lower MANSA scores in the Balkan sample only. High levels of education seem to be less protective for refugees resettled in the West, possibly linked to migration-related challenges such as restrictive asylum legislation and need for language competence. Worse SQOL scores in older Balkan residents could be a reflection of greater difficulties in rebuilding their lives after the war. There may be fewer opportunities for older people at the time of substantial political and socio-economic changes in the region. Previous studies have reported similar findings in refugee samples [10,30].

Posttraumatic stress symptoms have been linked with reduced SQOL measured on MANSA in both non-war [31] and war-affected populations [10], and this association may persist for many years after the actual traumatic events. Other QOL studies carried out in clinical and community samples of refugees and war veterans obtained similar results [9,32-35]. Our findings are consistent with these reports. Almost a decade after the occurrence of traumatic war events, posttraumatic stress symptoms still had a strong negative impact on SQOL in both groups. Amongst Balkan residents greater exposure to war events was uniquely associated with lower SQOL, even after controlling for posttraumatic stress symptoms and non-war trauma. This finding is consistent with other reports on the long-term impact of traumatic war events on QOL [10,30,36]. However, in refugees currently living in the Western countries this association was no longer significant in the multivariate analysis. This may be due to the fact that adaptation in resettlement involves a complex interplay of different elements of pre- and post-migration experience [37]. Indeed, experiencing a greater number of migration-stressors was negatively associated with SQOL in the refugee sample.

Overall, our findings indicate the importance of the post-war environment for both refugees in the West and those who stayed in the Balkans. Unemployment was associated with lower MANSA scores in both groups, even after traumatic events and posttraumatic stress were taken into account. Similar findings were reported for the community sample of war-exposed people from the same region [10], but also for mental health patients assessed in the post-war period [29]. In a 10 year longitudinal study on tortured refugees in Denmark, unemployment at follow-up was an important predictor of low quality of life [38]. Living in collective accommodation had a negative impact on SQOL in the Balkan sample, consistent with previous findings from the region [10], but also from other post-conflict settings [39]. Being married and reporting recent meetings with friends played a protective role for both groups, thus emphasising the importance of social contacts for subjective appraisal of life conditions. This again is similar to findings from other studies on QOL in both refugees and those who remained in post-conflict settings [30,38,40].

### Implications

The study has implications for policy and practice of programmes to mitigate effects of traumatic war events and improve well-being in both people residing in post-conflict regions and amongst refugees in the West. Our findings suggest that, in addition to treating symptoms, aid programmes should consider a broader range of interventions aimed at improving social outcomes such as employment, living conditions and social networks. They lend support to calls to develop new, integrated therapeutic approaches that take into account the social context of recovery [11,41-43]. Furthermore, improving SQOL by addressing current socio-economic stressors may lead to improvement in war-related PTSD symptoms which are known to be difficult to treat [43,44]. A longitudinal study carried out within the same project has established a bi-directional association between hyperarousal PTSD symptom cluster and SQOL [45].

## Conclusion

The question as to whether symptoms or social factors are important for SQOL when both are considered in the same analysis has found a clear answer in this study, i.e. that both are relevant and that their impacts are to some extent independent of each other. Almost a decade after the war in the former Yugoslavia, posttraumatic stress symptoms as well as socio-economic aspects of the post-war environment had an impact on SQOL. Future research may explore how interventions to address post-traumatic stress symptoms and adverse social factors can be combined to improve the SQOL of war affected populations.

## Competing interests

The authors declare that they have no competing interests.

## Authors' contributions

MB, SP, DA, TF, AK, GMG, DLT, NM, MP, MS, AM all made substantial contributions to the design of the study and data collection. AM and DG conducted data analysis. AM, DG, MB, SP, DA, TF, AK, GMG, DLT, NM, MP and MS all contributed to interpretation of the findings and critical revision of drafts. All authors read and approved the final manuscript.

## Pre-publication history

The pre-publication history for this paper can be accessed here:

http://www.biomedcentral.com/1471-2458/13/624/prepub
